# Extracellular NAD^+^ levels are associated with CD203a expression on Th17 cells and predict long-term recurrence-free survival in hepatocellular carcinoma

**DOI:** 10.1007/s00432-025-06155-4

**Published:** 2025-03-19

**Authors:** Julia Babigian, Philipp Brunnbauer, Can Kamali, Sebastian Knitter, Eriselda Keshi, Matthäus Felsenstein, Philipp Haber, Isis Lozzi, Wenzel Schöning, Johann Pratschke, Felix Krenzien

**Affiliations:** 1 Department of Surgery, Campus Charité Mitte and Campus Virchow-Klinikum, Charité-Universitätsmedizin, Corporate Member of Freie Universität Berlin, Humboldt-Universität zu Berlin, and Berlin Institute of Health, Augustenburger Platz 1, 13353 Berlin, Germany; 2 Berlin Institute of Health (BIH), 10178 Berlin, Germany; 3 Institute of Legal Medicine and Forensic Sciences, Charité-Universitätsmedizin Berlin, Corporate Member of Freie Universität Berlin, Humboldt-Universität zu Berlin, Turmstraße 21, 10559 Berlin, Germany

**Keywords:** Hepatocellular cancer, Liver fibrosis, NAD^+^, Th17 cells, CD203a

## Abstract

**Background and aims:**

Mortality rates for hepatocellular carcinoma (HCC) remain high, while multimodal treatment approaches offer new perspectives. Here, we investigated the association of extracellular nicotinamide adenine dinucleotide (eNAD^+^) on ecto-nucleotide pyrophosphatase/phosphodiesterase 1 (CD203a, ENPP1 or PC-1) on Th17 cells in relation to the likelihood of HCC recurrence following liver resection.

**Method:**

The study compared heparinized blood plasma samples from 95 patients who underwent liver resection, including 25 patients with HCC and 24 control patients without liver disease. Plasma eNAD^+^ concentrations were determined using a heat-based dichotomous pH extraction method, followed by enzymatic cycling and a colorimetric assay for quantification. Fibrosis was graded histologically using the Desmet score (F0–F4). Surface expression analysis was performed using flow cytometry.

**Results:**

With increasing grades of liver fibrosis predominant in HCC patients, a significant reduction in plasma eNAD^+^ concentrations was measured (p < 0.05). Further, a significant correlation was found between HCC patients and CD203a expression on CD4^+^, CCR4^+^ as well as CCR6^+^ T cells (p < 0.05). Patients who exhibited high proportions of CD203a expressing Th17 cells (CD4^+^, CCR6^+^ CCR4^+^) post surgery were found to be at a sixfold increased risk (HR 6.38, 95% Cl 1.51–27.00) of HCC recurrence and had a median recurrence-free survival of 233 days (p < 0.05), compared to patients with low CD203a expressing Th17 cells (CD4^+^ CCR6^+^ CCR4^+^). Similarly, patients who had a high proportion of CD203a expressing Th17 cells (CD4^+^ CCR6^+^) following surgery had a fivefold increased risk (HR 5.56, 95% Cl 1.58–19.59) of HCC recurrence and a median recurrence-free survival of 334 days (p < 0.05) compared to those with low CD203a expressing Th17 cells (CCR6^+^).

**Conclusion:**

The data indicates that eNAD^+^ levels are decreased in patients with liver fibrosis or cirrhosis. Strikingly, patients with high CD203a expression on Th17 cells had a significantly increased likelihood of recurrence, highlighting its potential as a valuable prognostic marker and a possible therapeutic target.

**Supplementary Information:**

The online version contains supplementary material available at 10.1007/s00432-025-06155-4.

## Introduction

High proliferation rates of liver cells in the context of liver cirrhosis and, in combination with DNA alterations, correlate with an increased prevalence of hepatocellular malignancies (Llovet et al. 2022). Generally, tumor stromata encompass a range of cellular elements including tumor cells, fibroblasts, myofibroblasts, vascular cells, and immune cells (Hanahan and Weinberg 2011; Hanahan and Coussens 2012). These processes contribute to the development of a tumor, as they maintain cellular signalling, promote unrestricted growth, hinder apoptosis, stimulate angiogenesis, and mitigate recognition by the immune system. The immune system of affected individuals has an important role in the malignant transformation, and is nowadays the target of various checkpoint inhibitors (for example PD-L1 or CTLA-4) as part of adjuvant or neoadjuvant therapeutic strategies for hepatocellular carcinoma (HCC) (Lee et al. 2020; Finn et al. 2020).

Chronic inflammation causes immunological changes, thereby enabling malignant tumor cells to evade immune cell surveillance utilising immune escape mechanisms (Yang et al. 2019). The tumor cells are protagonists of such processes as they generate an immunosuppressive tumor microenvironment (TME), which hinders the immune system's ability to coordinate antitumor immune responses, thereby impeding the ability to effectively combat tumor growth and progression (Khalil et al. 2016; Mahoney et al. 2015; Atanasov et al. 2019, 2020).

Nicotinamide adenine dinucleotide (NAD^+^) may play a pivotal role in immunomodulation of HCC. In previous work, we demonstrated that the concentration of extracellular NAD^+^ (eNAD^+^) in plasma is approximately 500 times lower than intracellular NAD^+^ concentrations in peripheral blood mononuclear cells (PBMCs) (Saqr et al. 2023). NAD^+^ can be moved via Connexin 43 (Cx43) hemichannels between the intracellular to extracellular compartment or as a result of apoptosis, hypoxia, inflammation, mechanical or chemical activation (Gasparrini et al. 2021). Moreover, eNAD^+^ can be metabolized via CD38, CD73, nucleotide pyrophosphatase/phosphodiesterase 1 and ADP-ribosyltransferases (ARTS) on the cell membrane (Gasparrini et al. 2021). Additionally, NAD^+^ has an influence on T cell populations and can modulate their differentiation (Elkhal et al. 2016).

CD203a (ectonucleotide pyrophosphatase or phosphodiesterase 1; ENPP1) is a type II transmembrane glycoprotein, which was initially discovered as a negative regulator of bone mineralization (Johnson et al. 1999, 2003). Therefore, it is expressed on mineralizing cells, such as osteoblasts and chondrocytes, but appears to be also localized on T cells (Ferretti et al. 2019). The functions of CD203a are diverse. In fact, the enzyme acts as a diphosphatase and diesterase, demonstrating extensive substrate specificity towards dinucleotides such as NAD^+^ and nicotinamide adenine dinucleotide phosphate (NADP^+^) in addition to triphosphates including adenosine triphosphate (ATP), uridine triphosphate (UTP), cyclic adenosine monophosphate (cAMP), and cyclic guanosine monophosphate-adenosine monophosphate (2′3′-cGAMP) (Goding et al. 2003; Li et al. 2015; Li et al. 2015; Namasivayam et al. 2017; Terkeltaub 2001). In this context, CD203a has the potential to utilise NAD^+^, Adenosine diphosphate ribose (ADPR) and ATP as substrates. Further, CD203a catalyzes 5′-phosphodiester bonds, predominantly using ATP as the substrate, resulting in the production of nucleoside 5′-monophosphates, mainly AMP. When CD203a processes NAD^+^ as the substrate, AMP and nicotinamide mononucleotide (NMN) serve as the reaction products. In contrast, if CD203a catalyses the substrate ADPR or ATP, the resulting main products are AMP and diphosphate (PPi) (Nikiforov et al. 2011).

Although there is mounting evidence of a relevant impact of purinergic signalling towards effective immune responses, research has not yet linked plasma NAD^+^ levels with CD203a expression on circulating Th17 cells in the plasma of HCC patients. Therefore, we investigated the influence of eNAD^+^ on CD203a on Th17 cells in relation to the likelihood of HCC recurrence following liver resection. These findings could pave the way for future targeted immunomodulatory therapies for HCC.

## Materials and methods

### General characteristics of the study

This prospective observational study was conducted under the approval of the Charité ethics committee (Ethikkommission der Charité Universitätsmedizin Berlin) under application numbers EA1/193/16, EA1/291/16, and EA1/018/17. The study recruited 95 patients between December 2016 and May 2018 at the Department of Surgery, Campus Virchow Klinikum (Charité—Universitätsmedizin Berlin). Inclusion criteria for the liver resection cohort were: age 18 years or older, not currently pregnant, indication for liver resection, written informed consent, and the ability to provide information and consent. In total, 25 patients suffered from HCC. The control group comprised 24 patients who underwent surgery, mostly abdominal hernia operations (supplement table I), respectively. The inclusion criteria for control patients were; absence of liver disease, no ongoing immunosuppressive therapy, no chronic infectious and no history of malignant tumors. Participants who did not fulfil these criteria were excluded from the study.

### Isolation and cryopreservation of peripheral blood mononuclear cells

PBMCs were obtained from HCC patients and control patients. Venous blood was collected aseptically before and several days after surgery, with 3 ml collected in an EDTA tube and 4 ml collected in a heparinized tube. The blood samples were subsequently processed for the preservation of plasma. The tubes were spun at 2500 g and 4 °C for 15 min. Subsequently, the blood plasma was transferred to fresh tubes, cryopreserved in liquid nitrogen, and ultimately kept at − 80 °C.

To isolate PBMCs, the absent blood plasma in the EDTA tube was substituted with 2 ml of FACS buffer [PBS (500 ml) + 1% BSA (5 g) + NaN₃ (0.5 g)], filtered with a 0.2 μm filter prior to usage. The EDTA blood sample was mixed with 40 ml of lysis buffer, comprising 80.2 g/l NH₄Cl (1.5 mol/l), 8.4 g/l NaHCO₃ (0.10 mol/l), and 372.2 mg/l EDTA dissolved in 800 ml sterile distilled H_2_O adjusted to pH 7.4 after filtering through a 0.2 µm filter. Following a lysis time of several minutes, the erythrocytes underwent lysis and the liquid became clear. Subsequently, the lysed cells were centrifuged at 400*g* and 4 °C for 5 min, and the supernatant was discarded. This process was repeated using 10 ml of lysis buffer. Remaining blood cells were temporarily stored on ice, and lysis was stopped by adding 2 ml of PBS (pH 7.4 phosphate-buffered saline solution containing 8.0 g NaCl, 0.2 g KCl, 1.42 g Na₂HPO₄, and 0.27 g KH₂PO₄ per liter). Finally, isolated PBMCs were placed in cryopreservation vials, each containing approximately 10,000,000 cells with 100 μl of DMSO and 900 μl of FBS serum (fetal bovine serum). The cryopreservation vials were kept in a refrigerated container (Thermo Scientific™ Mr. Frosty™) at − 80 °C for 24 h prior to being transferred for long-term storage at − 156 °C.

### Thawing of peripheral blood mononuclear cells

To defrost the cryopreserved PBMCs, first 1 ml of CTL Anti-Aggregate Wash Medium™ for each sample in a 37 °C water bath was thawed. Then, it was mixed with 19 ml of T-cell medium (RPMI 1640, supplemented with 5 ml of l-alanine/l-glutamine, 4 µl of β-Mercaptoethanol, 5 ml of Penicillin/Streptomycin (100 U/ml), and 50 ml of FBS). The solution was divided into two Falcon tubes, with 10 ml in each and incubated in a water bath for 20 min to regulate the pH and temperature. The cryopreserved samples were held in a water bath of 37 °C for 1 min to partially thaw them while keeping a frozen portion intact. Following this, the nearly thawed PBMC were carefully mixed with 1 ml of CTL Anti-Aggregate Wash Medium and slowly transferred dropwise into the remaining solution of CTL and RMPI medium in a Falcon tube. The cell suspension was centrifuged at 400*g* for 10 min at room temperature. The supernatant was subsequently removed, and the remaining 10 ml of solution were subjected to the same process. The supernatant was then removed, and the remaining 10 ml of solution were subjected to the same process. Thereafter, cell counting was performed. The supernatant was removed once again, and the PBMCs were suspended in 300 μl of EasySepCell™ Medium, which is a mixture of Dulbecco's PBS, FBS (2%), and EDTA (1 mM) in PBS from STEMCELL Technologies. The supernatant was then removed, and the remaining 10 ml of solution were subjected to the same process.

### Isolation and cultivation of T cells

For the purpose of T cell isolation, the EasySep™ Human T Cell Isolation Kit [(Catalog #17951) STEMCELL Technologies GmbH, Cologne, Germany] was utilized. This kit employs antibody complexes conjugated with magnetic particles to effectively isolate unwanted cells. Initially, 250 μl of thawed cells were mixed with 12 μl of an isolation cocktail, and the solution was incubated in FACS tubes for 5 min at room temperature. Following this step, 10 μl of RapidSpheres were added, and the whole suspension was filled up to a total volume of 2.5 ml with EasySep™ Medium. Next, the FACS tubes were placed in the EasySep™ magnet [(Catalog #18000] STEMCELL Technologies GmbH, Cologne, Germany) for three minutes to isolate T cells.

Eventually, isolated T cells were then carefully transferred to a new FACS tube, and a maximum of 1,000,000 isolated T cells, but at least 500,000, were transferred to a 96-well standard microtiter plate. The cells were stained using anti-CD28 and protein transport inhibitor (0.2 μl in 100 μl PBS). The plate was incubated with T cell medium replenished to 100 μl for four hours at 37 °C.

### Antibody staining

After four hours of incubation, the T cells that were isolated underwent washing with 100 μl of PBS and were centrifuged at room temperature for 5 min with 400 g. The supernatant was removed meticulously, and cells were stained with extracellular stains (50 μl each of 5 μl of PBS, 5 μl of FC block, 1 μl of CD203a, 1 μl of CCR6, 1 μl of CCR4, and 0.1 μl of Zombie UV) for 15 min in a dark at room temperature. Following, the cells were centrifuged with 100 μl of FACS buffer for 5 min at 400*g* and room temperature. The supernatant was subsequently removed before the cells were treated with 100 μl of fixation buffer for 20 min.

Afterward, intracellular staining was performed using 48 μl of Perm Wash, 1 μl each of IL-17 and IL-22, with the cells again being centrifuged with 100 μl of Perm Wash and stained with appropriate antibodies for 15 min. The cells were washed with 100 μl of FACS buffer, centrifuged, and then replenished with 200 μl of FACS buffer. The cells were finally transported on ice for flow cytometric measurement using the Hoechst UV filter and BD LSRFortessa™ X-20, with the FACSDiva software BD FACSDiva™ v9.0 (BD Biosciences, San Jose, USA). A number of events adapted to the patient sample were measured in each case. To adjust for overlapping emission spectra during multiple staining events, we utilized the FACSDiva software to calculate the compensation matrix. To establish compensation, we conducted a single staining on all antibodies in patient samples from the group who had undergone liver resection.

### eNAD⁺ measurement in plasma

eNAD^+^ levels were measured in all 95 patients of the liver resection group and in 24 control patients pre- and postoperatively. To determine NAD⁺ levels, a dichotomous pH extraction procedure incorporating heat was employed as previously described (Brunnbauer et al. 2018).

Frozen heparin plasma was thawed gradually at ambient temperature, then partitioned into two equal portions—one for NAD⁺ extraction and the other mixed with 270 μl of an albumin adjusted revised simulated body fluid (r-SBFA) for NADH extraction. The r-SBF solution was prepared by adjusting the pH to 7.4 in 1000 ml of DEPC water. The ingredients used were sodium chloride (5.403 g), sodium hydrogen carbonate (0.740 g), sodium carbonate (2.046 g), potassium chloride (0.225 g), potassium dihydrogen phosphate (0.138 g), magnesium chloride hexahydrate (0.311 g), 2-(4-(2-hydroxyethyl)−1-piperazinyl)ethanesulfonic acid (HEPES) (11.928 g), calcium chloride dihydrate (0.388 g), sodium sulfate (0.072 g) and bovine serum albumin (40 g).

To extract the samples, acid and base solutions were used as follows: 300 μl of 0.3 N HCl for eNAD^+^, 300 μl of 0.3 N KOH for NADH, and 300 μl of 0.3 N HCl for r-SBFA. The tubes were then incubated at 60 °C for 10 min, followed by equilibration on ice for 10 min. After 300 μl of neutralization buffer was added and centrifugation was performed at 16,000*g* for 10 min at 4 °C, a transparent 96-well standard microtiter plate was used for sample measurement. For the measurement of eNAD⁺, 50 μl of each sample were utilized, whereas for the measurement of NADH, 5 μl of each sample and 45 μl of a control sample (300 μl of r-SBF in 1:10 dilution) were utilized. Then, 150 μl of the master mix [TEA buffer: 1:10 diluted and adjusted to a pH of 7.4, alcohol dehydrogenase (ADH): 1:10 diluted, polymethylsiloxane polyhydrate (PMS): 10 mg/ml solution, 3-(4,5-dimethylthiazol-2-yl)−2,5-diphenyltetrazolium bromide (MTT)] was mixed with the samples. A solution of 1 mg/ml diluted in 100% ethanol (EtOH) was resuspended twice following timed conditions. After leaving it to stand for 5 min at room temperature in darkness, the microplate was analysed using a microplate reader (Infinite 200 PRO, Tecan Trading AG, Switzerland) at a temperature of 25 °C to determine the absorbance at 565 nm for 30 min.

### Statistics

The statistical analysis was conducted using GraphPad Prism (GraphPad Software, Version 6.01, San Diego, USA). Patient demographics and clinical data were summarized using mean values and standard deviations. Unpaired t-tests were used for continuous variables, and chi-square tests were used for categorical variables. To assess the respective datasets for normality and warrant the use of unpaired t-tests, the D'Agostino–Pearson Omnibus Test was employed. Differences between liver resection patients and controls were evaluated using one-way ANOVA for normally distributed variables. The study compared the preoperative proportions of various subtypes of Th17 cells expressing CD203a in patients with HCC and healthy individuals using unpaired t-tests. Receiver operating characteristic (ROC) curves were used to evaluate diagnostic and prognostic biomarkers, with the area under the curve (ROC-AUC) measuring their diagnostic accuracy. To determine the optimal threshold for the potential biomarkers, the Youden index was derived. Recurrence-free and overall survival probabilities were calculated through implementation of the Logrank (Mantel–Cox) and Gehan-Breslow-Wilcoxon tests and conducted specifically in the HCC subgroup. Additionally, the Hazard Ratio (Mantel–Haenszel and Logrank) was computed. Statistical significance was determined using an alpha value of p < 0.05.

## Results

### The concentration of NAD^+^ depends on fibrosis severity and liver tumor type

**Fig. 1:**
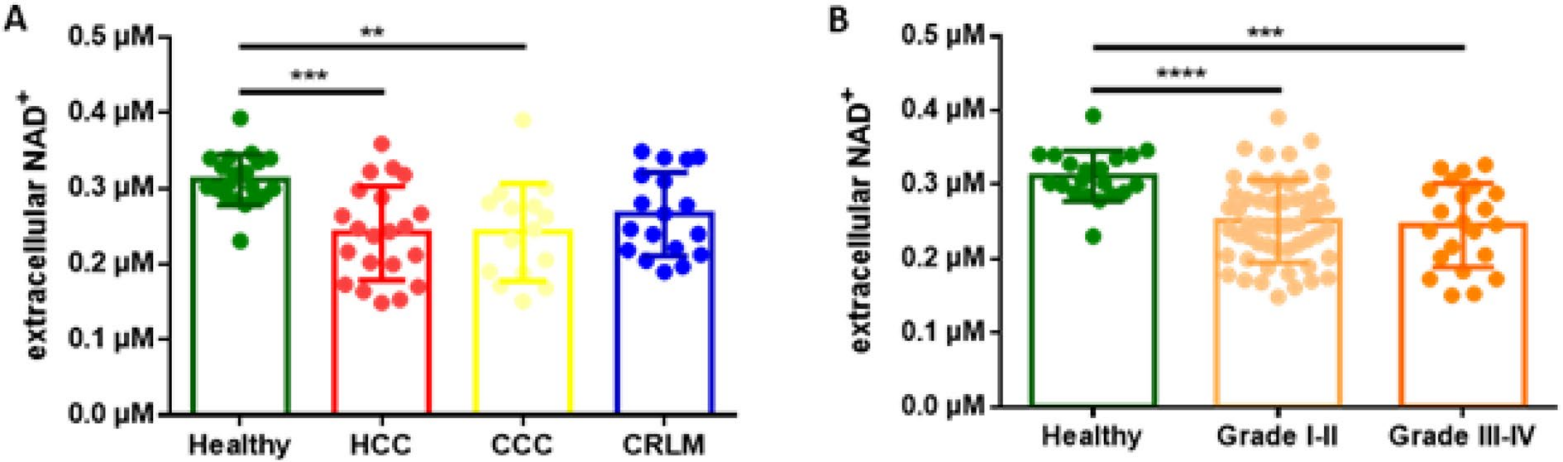
Preoperative eNAD+ concentration in patients with HCC (n = 25), CCC (n = 23), CRLM (n = 46), Fibrosis grades I–IV (I + II n = 58, III + IV n = 27) and healthy controls (n = 24), scatter-dot plot, mean with SD. **A** Preoperative eNAD^+^ concentration in patients with HCC, CCC, CRLM and healthy controls. **B** Preoperative eNAD^+^ concentration in patients with fibrosis grades I–II, III–IV and healthy controls, statistics: oneway ANOVA with confidence level of CL = 95%, applying a significance level of p < 0.05; *p ≤ 0.05, **p ≤ 0.01, ***p < 0.001, ****p < 0.0001

The concentration of eNAD^+^ was determined in heparin plasma samples from the previously mentioned patient cohorts in accordance with the established protocol. The pre-operative plasma concentration of eNAD^+^ for patients with HCC was measured significant to be lower than the control group (Fig. 1A). Additionally, a decreased blood eNAD^+^ concentration was observed in patients with cholangiocellular carcinoma (CCC) (Fig. 1A). No significant difference was found in patients with colorectal liver metastasis (CRLM) compared to the control group (Fig. 1A). Grade of fibrosis was assessed according to Desmet (1997). The eNAD^+^ concentration in the blood of stage I/II and III/IV patients was significantly reduced when compared to the healthy control group (Fig. 1B). No significant difference was found between grade I/II and III/IV of fibrosis.

We observed that eNAD^+^ levels exhibited a notable decrease, particularly among patients with HCC, as compared to the healthy control group. This decline was most likely associated with the presence of liver fibrosis or cirrhosis.

### Th17 cells expressing CD203a are reduced in patients with HCC

**Fig. 2:**
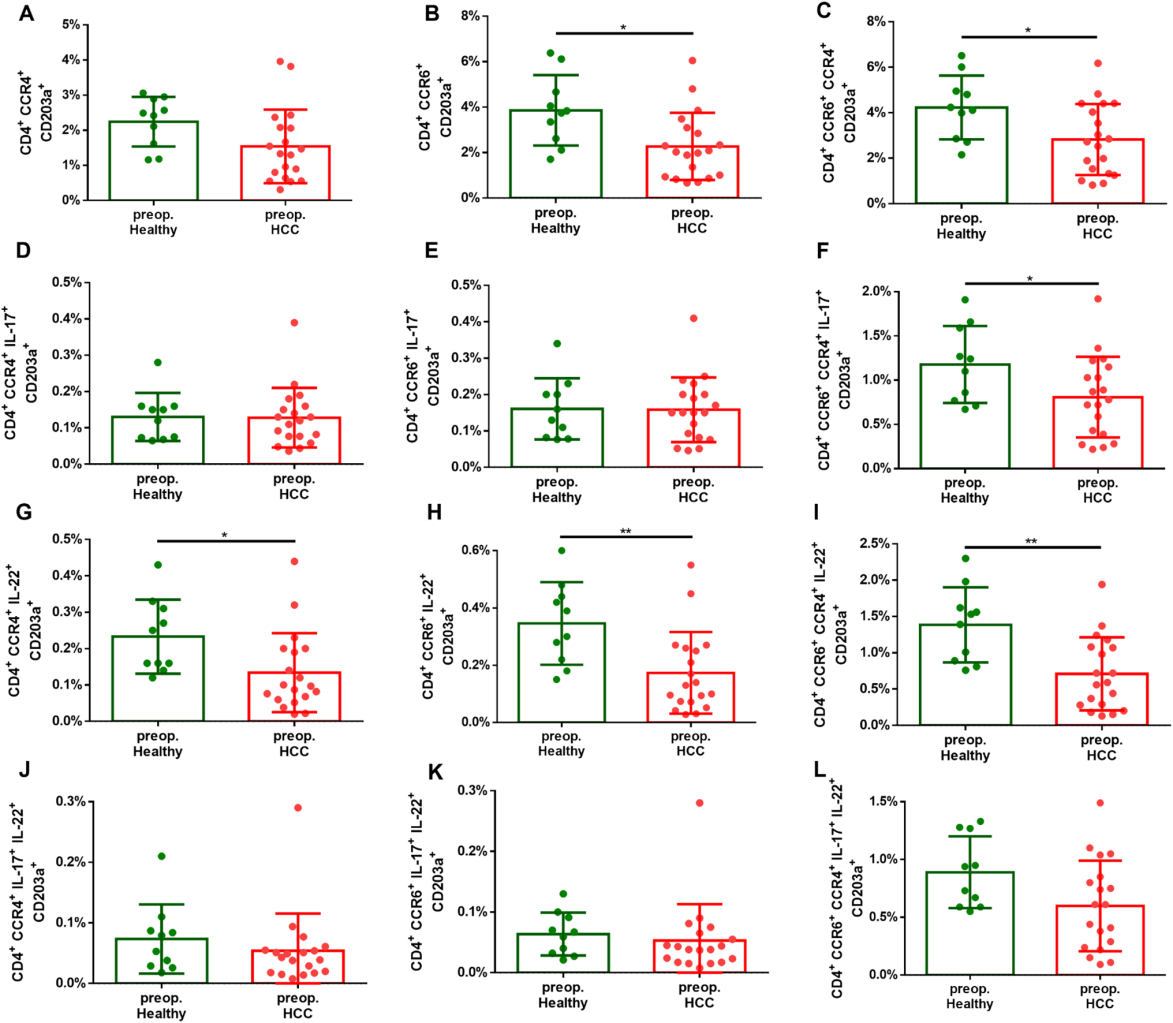
Preoperative fractions of the various subtypes of Th17 cells (CD4^+^ CCR6^+^ CCR4^+^ IL-17^+^ IL-22^+^) with CD203a expression in HCC (n = 20) and healthy patients (n = 10), Scatter-dot plot, Mean with SD **A** preoperative proportion of Th17 cells (CD4^+^ CCR4^+^) with CD203a expression, **B** preoperative proportion of Th17 cells (CD4^+^ CCR6^+^) with CD203a expression, **C** preoperative proportion of Th17 cells (CD4^+^ CCR4^+^ CCR6^+^) with CD203a, **D** preoperative proportion of Th17 cells (CD4^+^ CCR4^+^ IL-17^+^) with CD203a expression, **E** preoperative proportion of Th17 cells (CD4^+^ CCR6^+^ IL-17^+^) with CD203a expression, **F** preoperative proportion of Th17 cells (CD4^+^ CCR4^+^ CCR6^+^ IL-17^+^) with CD203a expression, **G** preoperative proportion of Th17 cells (CD4^+^ CCR4^+^ IL-22^+^) with CD203a expression, **H** preoperative proportion of Th17 cells (CD4^+^ CCR6^+^ IL-22^+^) with CD203a expression, **I** preoperative proportion of Th17 cells (CD4^+^ CCR4^+^ CCR6^+^ IL-22^+^) with CD203a expression, **J** preoperative proportion of Th17 cells (CD4^+^ CCR4^+^ IL-17^+^ IL-22^+^) with CD203a expression, **K** preoperative proportion of Th17 cells (CD4^+^ CCR6^+^ IL-17^+^ IL-22^+^) with CD203a expression, **L** preoperative proportion of Th17 cells (CD4^+^ CCR4^+^ CCR6^+^ IL-17^+^ IL-22^+^) with CD203a expression; statistics: unpaired-t-test with confidence level of CL = 95%, applying a significance level of p < 0.05; *p ≤ 0.05, **p ≤ 0.01, ***p < 0.001, ****p < 0.0001

**Fig. 3:**
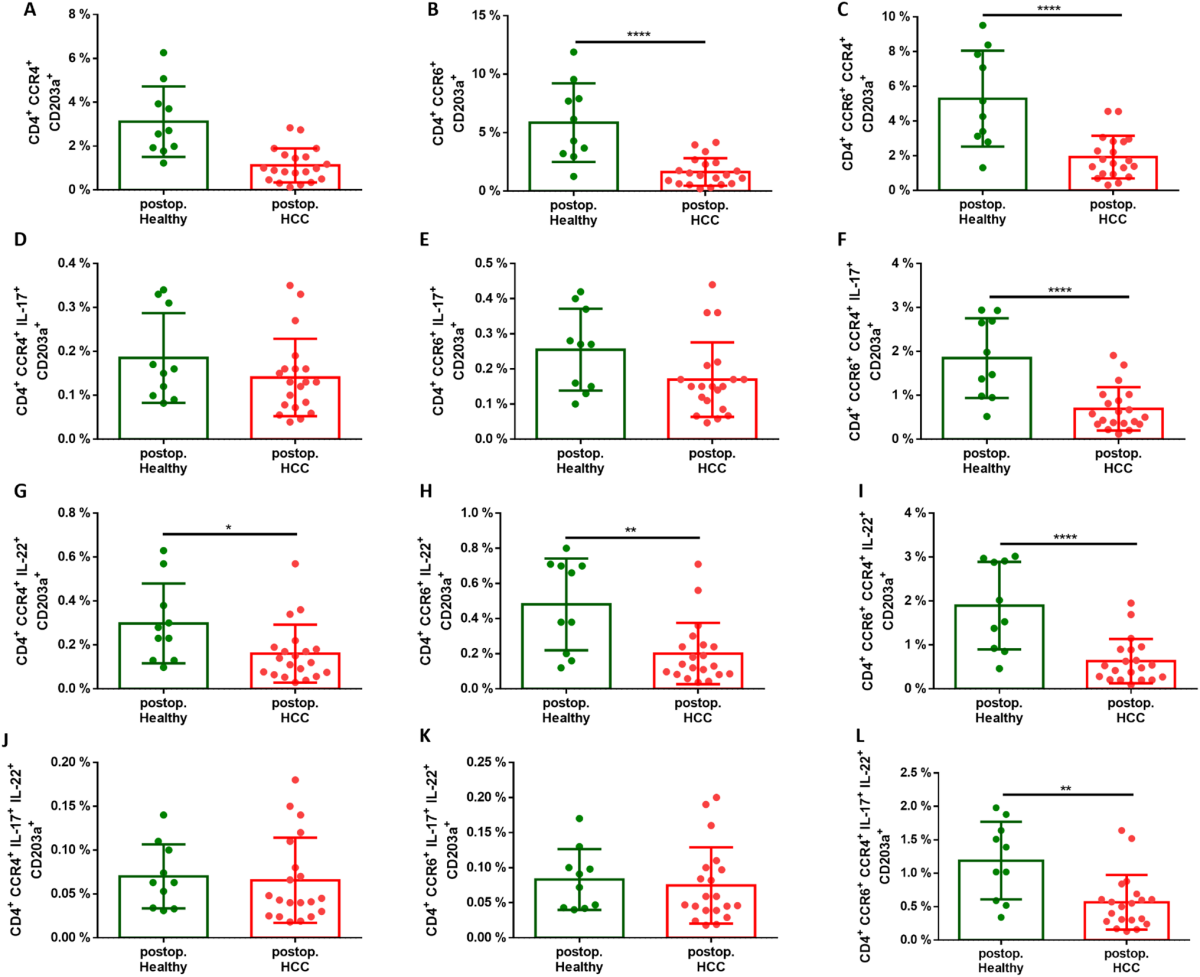
Postoperative fraction of the various subtypes of Th17 cells (CD4^+^ CCR6^+^ CCR4^+^ IL-17^+^ IL-22^+^) with CD203a expression in HCC (n = 20) and healthy patients (n = 10), scatter-dot plot, mean with SD **A** Postoperative proportion of Th17 cells (CD4^+^ CCR4^+^) with CD203a expression, **B** Postoperative proportion of Th17 cells (CD4^+^ CCR6^+^) with CD203a expression, **C** postoperative proportion of Th17 cells (CD4^+^ CCR4^+^ CCR6^+^) with CD203a, **D** postoperative proportion of Th17 cells (CD4^+^ CCR4^+^ IL-17^+^) with CD203a expression, **E** postoperative proportion of Th17 cells (CD4^+^ CCR6^+^ IL-17^+^) with CD203a expression, **F** postoperative proportion of Th17 cells (CD4^+^ CCR4^+^ CCR6^+^ IL-17^+^) with CD203a expression, **G** postoperative proportion of Th17 cells (CD4^+^ CCR4^+^ IL-22^+^) with CD203a expression, **H** postoperative proportion of Th17 cells (CD4^+^ CCR6^+^ IL-22^+^) with CD203a expression, **I** postoperative proportion of Th17 cells (CD4^+^ CCR4^+^ CCR6^+^ IL-22^+^) with CD203a expression, **J** postoperative proportion of Th17 cells (CD4^+^ CCR4^+^ IL-17^+^ IL-22^+^) with CD203a expression, **K** postoperative proportion of Th17 cells (CD4^+^ CCR6^+^ IL-17^+^ IL-22^+^) with CD203a expression, **L** postoperative proportion of Th17 cells (CD4^+^ CCR4^+^ CCR6^+^ IL-17^+^ IL-22^+^) with CD203a expression; statistics: unpaired-t-test with confidence level of CL = 95%, applying a significance level of p < 0.05, *p < 0.05, **p < 0.01 and ***p < 0.001, ****p < 0.0001

Following T cell stimulation and culture, Th17 cells were identified by measuring CD4^+^ T cells expressing the cytokines IL-17 and/or IL-22, along with the surface markers CCR6^+^ and/or CCR4^+^ via cytometric analysis. Comparisons were made between the levels of Th17 cells expressing CD203a before (Fig. 2) and after (Fig. 3) surgery in patients with HCC and healthy controls.

The fraction of Th17 cell population was decreased in patients with HCC when compared to healthy controls, both pre- and postoperatively. Similarly, among the subset of Th17 cells expressing CCR6^+^, but lacking interleukin expression also exhibited significantly lower levels in patients with HCC, both pre- and postoperatively. In addition, lower frequencies in HCC patients have been observed in all subpopulations of Th17 cells (CCR6^+^ and CCR4^+^), independent of interleukin expression.

### Influence of CD203a-expressing Th17 cells on recurrence-free and overall survival following liver resection for HCC

**Fig. 4:**
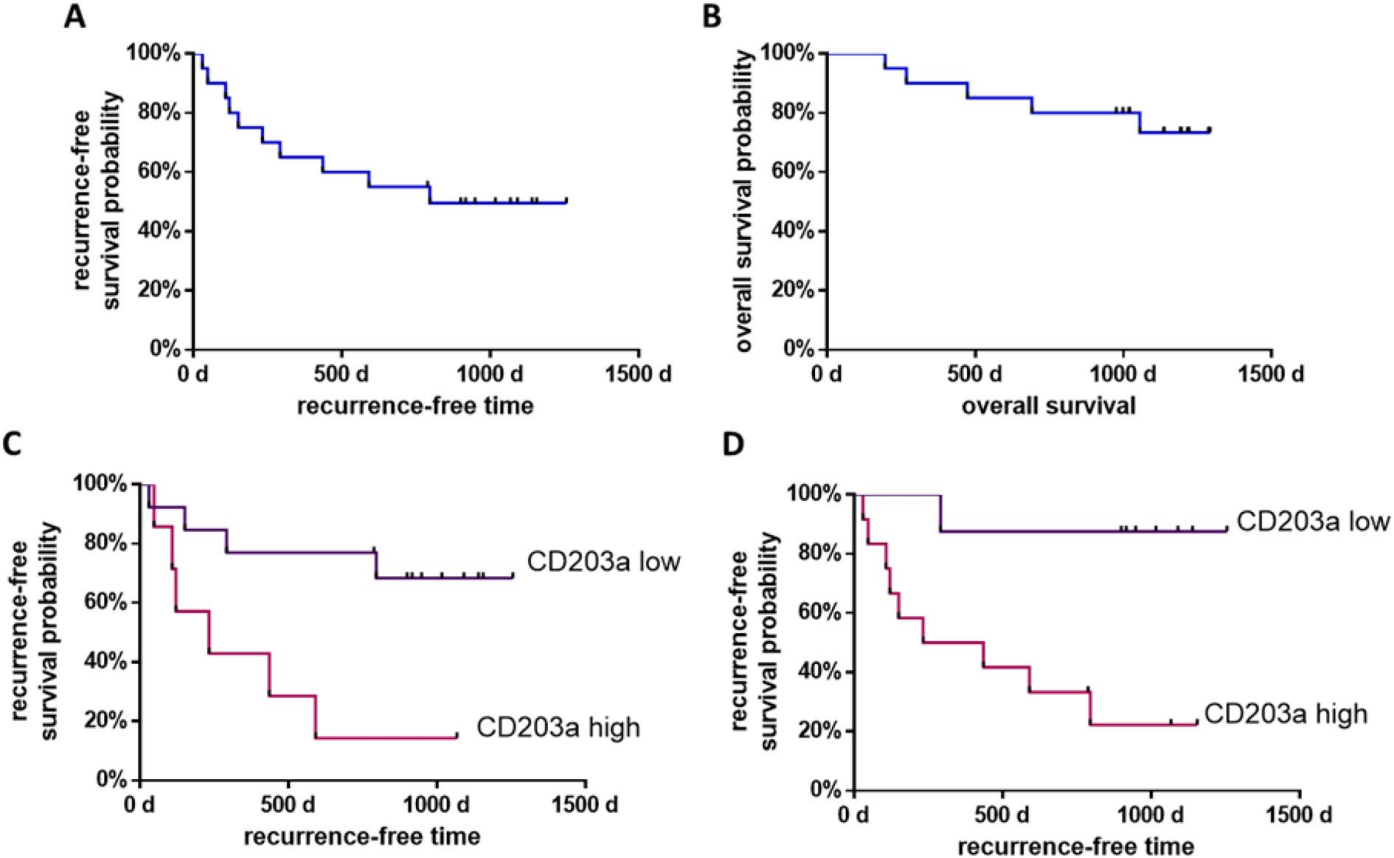
Recurrence-free and overall survival probability of patients in cohort LR (n = 20), Kaplan Meier curves. **A** Recurrence-free survival probability, median survival 796 days; **B** overall survival probability; **C** recurrence-free survival probability of proportion of CD203a expressing Th17 cells (CCR6^+^ CCR4^+^), *p < 0.05; **D** recurrence-free survival probability of proportion of CD203a expressing Th17 cells (CCR6^+^), **p < 0.01; statistics: log rank (Mantel–Cox), Gehan–Breslow–Wilcoxon test and hazard ratio (Mantel–Haenszel and log rank)

During the study period, 10 patients (50%) who underwent liver resection experienced local liver recurrences, while 10 patients (50%) (n = 10) remained recurrence-free at the last MRI follow-up. The most prolonged recurrence interval was observed 796 days after the operation, while the mean recurrence time measured 208 days (SD: 253 days). The longest period of survival without documented recurrence was 3.4 years (1256 days) following liver resection. The median survival period without documented recurrence was 2.1 years (796 days) (see Fig. 4A).

Subsequently, the overall survival probability was assessed in the liver resection patient group. During the observation period, five deaths occurred. The earliest death was recorded 196 days post-surgery, while the latest occurred 1055 days after the operation, with a mean survival time of 536 days (SD 348 days). Note, due to the small number of individuals who died, the median survival time could not be estimated (see Fig. 4B).

Patients with a postoperatively high proportion of CD203a expressing Th17 cells (CD4^+^ CCR6^+^ CCR4^+^) showed a sixfold increased risk (HR 6.38, 95% Cl 1.51–27.00) with of HCC recurrence and a median recurrence-free survival of 233 days (Fig. 4C), compared to CD203a Th17 cells with low expression (CCR6^+^ CCR4^+^). Similarly, patients with a postoperative high proportion of CD203a expressing Th17 cells (CD4^+^ CCR6^+^) had a fivefold increased risk (HR 5.56, 95% Cl 1.58–19.59) of recurrence and a median recurrence-free survival of 334 days (Fig. 4D), compared to low CD203a expressing Th17 cells (CCR6^+^). The cut-offs are shown in Table 1.

**Table 1:** Prognostic significance of CD203a-expressing Th17 Cells (CCR6+ CCR4+) for HCC recurrence: this table presents the threshold values, sensitivity, specificity, Youden index, ROC AUC, and p-values for the prognostic biomarkers associated with the proportion of CD203a-expressing Th17 cells in predicting the recurrence of hepatocellular carcinoma (HCC)

	Cut-off (%)	Sensitivity (%)	Specificity (%)	Youden-index	ROC-AUC	p-Value
Proportion of CD203a expressing Th17 cells (CCR6^+^ CCR4^+^)	81.05	60	90	0.50	0.77	0.04
Proportion of CD203a expressing Th17 cells (CCR6^+^)	38.10	90	70	0.60	0.81	0.01

### Evaluation of CD203a as a prognostic and diagnostic biomarker

To evaluate CD203a as a biomarker for predicting outcomes in patients with HCC, we compared CD203a levels in preoperative HCC patients with those in a healthy control group.

Tables 1 and 2 presents the determined cut-off values as well as sensitivity, specificity, ROC-AUC, and Youden index for prognostic and diagnostic biomarkers predicting recurrence.

**Table 2:** Diagnostic significance for the occurrence of HCC of the NAD^+^ concentration and the number of Th17 cells (CCR6^+^ CCR4^+^ IL-22^+^) with CD203a expression; threshold, sensitivity, specificity, Youden index, ROC AUC and p-value of diagnostic biomarker

	Cut-off	Sensitivity (%)	Specificity (%)	Youden-index	ROC-AUC	p-Value
eNAD^+^ concentration	0.26 μM	77	90	0.67	0.81	0.007
Th17 cells (CCR4^+^ CCR6^+^ IL-22^+^) with CD203a expression	0.74%	63	100	0.63	0.83	0.003
CD4^+^ T cells with CD203a expression	13.25%	63	90	0.53	0.78	0.01
Th1 cells (CD4^+^ IFN-γ^+^) with CD203a expression	8.83%	94	70	0.53	0.90	0.0004
CD8^+^ T cells with CD203a expression	8.00%	94	80	0.74	0.93	0.0001
CTL (CD8^+^ IFN-γ^+^) with CD203a expression	3.00%	94	90	0.84	0.96	< 0.0001

The percentage of Th17 cells (CCR6^+^ CCR4^+^) expressing CD203a demonstrated high specificity at a cut-off value of 81.05%, particularly in identifying patients without recurrence. The corresponding ROC-AUC was at 0.77. Furthermore, Th17 cells (CCR6^+^) expressing CD203a exhibited a significant ROC-AUC of 0.81 at a cut-off value of 38.10%, indicating their potential as prognostic biomarkers for predicting recurrence. (Table 1).

For diagnostic biomarkers, we identified threshold values for sensitivity, specificity, and ROC-AUC (Table 2). The results demonstrated that Th17 cells (CCR4^+^ CCR6^+^ IL-22^+^) expressing CD203a exhibited 100% specificity at a cut-off of 0.74%, while eNAD^+^ concentration showed 90% specificity at a cut-off of 0.26 μM. The sensitivity levels were 63% for Th17 cells and 77% for eNAD^+^, with corresponding ROC-AUC values of 0.83 and 0.81. These findings indicate that decreased plasma NAD^+^ levels and reduced Th17 cell fractions with CD203a expression may serve as diagnostic markers for HCC.

## Discussion

Mortality rates for HCC remain high, while multimodal treatment approaches offer new perspectives for patients. Here, we investigated the influence of eNAD^+^ on CD203a on Th17 cells in relation to the likelihood of HCC recurrence after liver resection. Strikingly, the data indicate that eNAD^+^ levels decrease in patients with liver fibrosis/cirrhosis and are correlated with the expression of the ectoenzyme CD203a on Th17 cells. Consequently, patients with high expression of CD203a on Th17 cells had a significantly increased likelihood of recurrence, indicating their potential as valuable prognostic markers and as a possible target for therapy.

CD203a and other ectoenzymes involved in purinergic signalling can modulate the TME by altering the availability of extracellular nucleotides and nucleosides. CD203a regulates extracellular nucleotide hydrolysis, which was observed to significantly decrease in the bloodstream of Th17 cells in patients with HCC undergoing liver resection when compared to the control group (Figs. 2, 3). At the same time, HCC patients and those with incipient or advanced liver cirrhosis exhibit a markedly reduced concentration of eNAD^+^ (Fig. 1). While CD203a (ENPP3) might not directly increase or decrease NAD^+^ levels, it could potentially influence extracellular nucleotide pools, which in turn might have downstream effects on cellular metabolism and signalling pathways, including those involving NAD^+^.

The results obtained from HCC patients showed a decreased expression of CD203a on T cells, which might be caused by a negative feedback regulation because of the reduced concentration of eNAD^+^. Interestingly, CD203a hydrolyses extracellular nucleotides such as ATP, AMP, and Adenosine diphosphate ribose, which is the downstream of CD38, which is at the same time highly expressed on T cells. Moreover, CD203a might also be possible to hydrolyse NAD^+^ directly (Horenstein et al. 2013). Therefore, the decrease in eNAD^+^ concentration among HCC patients might lead to a decreased expression of CD203a. Further research at additional pre- and postoperative measurement time points and during the progression of liver cirrhosis could elucidate the temporal pattern of ectoenzyme expression and the evolution of eNAD^+^ concentration and ascertain whether their measurement enhances diagnostic and prognostic accuracy.

While CD203a might not directly increase or decrease eNAD^+^ levels, it could potentially influence extracellular nucleotide pools, which in turn might have downstream effects on cellular metabolism and signalling pathways, including those involving NAD^+^. Our data indicate an association between eNAD^+^ and CD203a expression. The potential influence of CD203a on NAD^+^ metabolism is complex and requires further mechanistic investigations. Animal models and functional assays are necessary to determine whether CD203a expression on Th17 cells directly modulates NAD^+^ levels or vice versa.

The observed lower preoperative eNAD^+^ levels in HCC and CCC patients compared to the control group may be due to impaired NAD^+^ metabolism associated with hepatocyte dysfunction, alterations in the tumor microenvironment, and local immunosuppressive processes specific to primary liver cancers. In contrast, CRLM patients may have relatively preserved liver function, which could explain why their eNAD^+^ levels were not significantly different from the control group.

Moreover, the reduced eNAD+ levels observed in patients with liver fibrosis or cirrhosis may be linked to impaired NAD^+^ biosynthesis, increased NAD^+^ consumption due to chronic inflammation, and enhanced enzymatic degradation by ectoenzymes such as CD38 or CD203a. Fibrotic livers have compromised metabolic capacity, which may reduce the systemic availability of NAD^+^ precursors, further contributing to decreased eNAD^+^ levels.

Th17 cells constitute a subtype of T helper cells that play a role in a number of pathological processes, including the development of malignant tumours, host defence, infections, autoimmune diseases and transplant rejection. While specific studies on CD203a expression in Th17 cells in the context of HCC is unknown, the role of CD203a in other cancers for example lung-, and breast cancer, suggests that it could be an important marker (Denniston et al. 2016; Ruiz-Fernández de Córdoba et al. 2022).

**Fig. 5:**
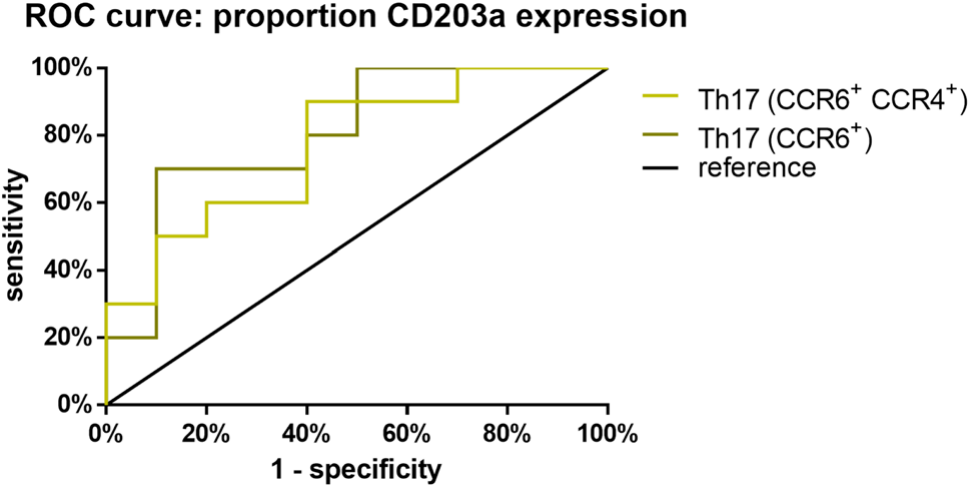
ROC curve for prognostic significance and recurrence-free survival probability of CD203a-expressing Th17 cells in cohort LR, ROC curve depicting the prognostic significance of the proportion of CD203a-expressing Th17 cells (CCR6^+^ CCR4^+^ and CCR6^+^). Statistics: the area under the ROC curve (AUC) is provided with a 95% confidence interval (CI), applying a significance level of p < 0.05 to reject the null hypothesis. Statistical significance is indicated by *p < 0.05

**Fig. 6:**
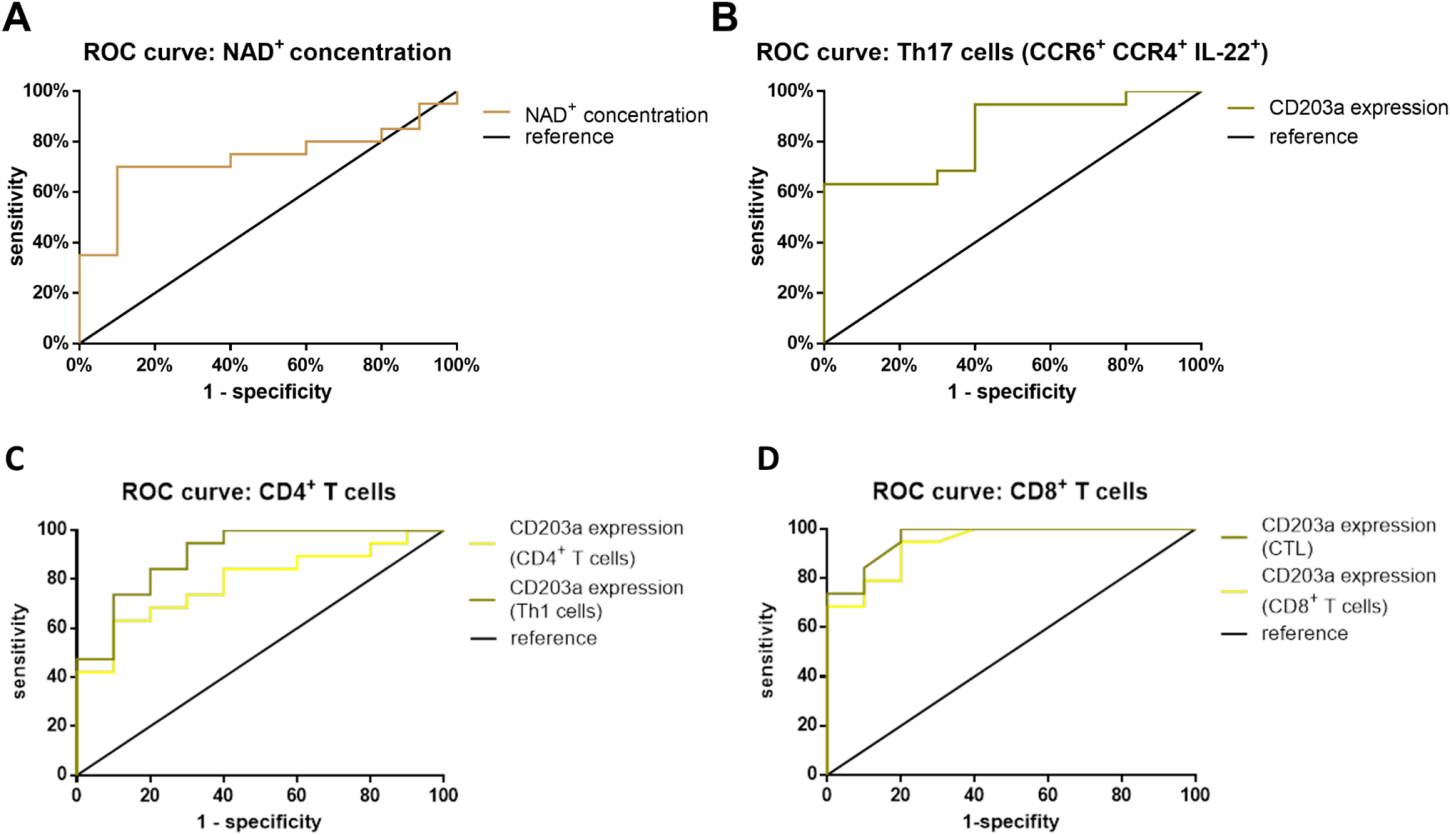
ROC curve for diagnostic significance of NAD^+^ concentration and Th17 cells with CD203a expression of patients with liver resection with reference line; **A** ROC curve of NAD^+^ concentration, **B** ROC curve of Th17 Cells (CCR6^+^ CCR4^+^ IL-22^+^), **C** ROC curve of CD4^+^ T cells and Th1 cells (CD4^+^ IFN-γ^+^), **D** CD8^+^ T cells and CTL cells (CD8^+^ IFN-γ^+^); statistics: AUC under the ROC curve with the confidence interval of CL = 95%, applying a significance level of p < 0.05 to reject the null hypothesis, *p < 0.05, **p < 0.01 and ***p < 0.001

Here, we demonstrated that the expression of ectoenzyme CD203a on Th17 cells may also have prognostic significance for HCC (Fig. 5). We have shown that an elevated percentage of Th17 cells that indicate CD203a were linked with a considerably decreased likelihood of the recurrence-free survival of liver resection patients. (Fig. 6C, D). This finding is unknown and underscores the importance of the immune system for HCC therapy. The role of CD203a as an immune modulator and potential immunotherapeutic target is becoming increasingly recognized, albeit it is premature to evaluate the clinical efficacy of CD203a-targeting agents. However, the results of phase 1 and 1/2 of ectonucleotidases inhibitors are forthcoming (Stagg et al. 2023).

Thorough evaluation of effective therapy requires objective assessment of patient survival and incidence of recurrences or metastases. The liver resection cohort patients received frequent outpatient follow-up care and abdominal MRI scans according to the German S3 guideline. The diagnosis of local recurrence, which was predominantly observed, transpired between the previous and subsequent appointment. This follow-up appointment was utilized to determine the occurrence of recurrence. In cases where no recurrence was detected, the date of the latest follow-up corresponds to the longest period of recurrence-free survival.

The quality assessment of a biomarker is crucial for its usage in diagnostics and assessing progression. The biomarker must differentiate between diseased and non-diseased individuals, determined using a cut-off value and ideally, have a correlation with the patient's stage of disease (Ruopp et al. 2008). The selection of the optimal cut-off value depends on its area of application. In the context of diagnosing HCC, an excessively high false negative rate may result in an adverse prognosis for the patient, as the diagnosis and subsequent therapy initiation occur too late. For the present study, the liver resection cohort was split into two subgroups depending on whether recurrence occurred or not. The Youden index was used to determine the best cut-off value, following the construction of a ROC curve. This study demonstrates that the presence of CD203a-expressing Th17 cells is associated with a five-fold increase in the risk of recurrence, indicating significant prognostic potential (Fig. 5). The ROC-AUC values for CD203a^+^ Th17 cells (CCR6^+^ CCR4^+^ and CCR6^+^) are 0.77 and 0.81, respectively. The Youden index values are 0.6 and 0.5. Thus, the potential prognostic biomarkers display acceptable distinguishability and moderate potential effectiveness in discerning between HCC patients with and without recurrence (Assmann et al. 1996). A diagnostic biomarker was sought for patients with HCC (Fig. 6). The eNAD^+^ concentration yielded a ROC-AUC of 0.81 and a Youden index of 0.67. Th17 cells, with CD203a expression (CCR4^+^ CCR6^+^ IL-22^+^), demonstrated a Youden index of 0.63 and an ROC-AUC of 0.83. The potential diagnostic biomarker's accuracy displayed excellent differentiability and the potential to effectively differentiate individuals with HCC from healthy individuals. However, a proper diagnostic accuracy study is needed because the current study design was purely exploratory.

In consideration of the methods employed, it is pertinent to note that a distinctive aspect of the sample processing was the measurement of the NAD^+^ concentration in the patients' serum. It is imperative that the blood samples be processed directly in the laboratory after the blood drawing to prevent any potential falsification of the measured concentration (Saqr et al. 2023). This is due to the fact that NAD^+^ can be hydrolysed or broken down by several enzymes present in the plasma. The stability of the plasma samples at room temperature was confirmed for a period of more than 30 min (Saqr et al. 2023; Brunnbauer et al. 2018).

The blood samples obtained for the study were collected over a period of 14 months. Following isolation, the PBMC of each sample were cryopreserved at − 156 °C for a period exceeding 12 months. This enabled the simultaneous measurement of the selected patient samples. Furthermore, all samples were treated in an identical manner. The cryopreservation and subsequent thawing of biological samples is an established procedure in clinical studies and is widely used in both biological and biomedical research. This process enables the long-term storage of samples while maintaining their functionality and viability (Germann et al. 2011). The most significant quality factors influencing the cryopreservation and thawing of PBMC are the standardized freezing and thawing protocol, the freezing method employed, and the length of cryopreservation (Tompa et al. 2018). It is acknowledged that despite the samples being stored in accordance with the required protocol, storage- and time-related changes may have occurred, which could potentially distort the overall result.

In the cryopreservation of T cells, the maintenance of functionality in the production of cytokines and interleukins represents a central objective of this study. The viability and functionality of T cells were demonstrated to be maintained in immunoassays after thawing when cryopreservation and continuous storage at below − 130 °C without temperature fluctuations were optimized (Angel et al. 2016). The second essential quality parameter is the duration of the T cell culture, which was four hours in this study. This is a pertinent factor in the recovery of the cells and the formation of cytokines and interleukins in this study. A T cell culture exceeding 12 h in duration results in a decline in viability and functionality when compared to measurements taken immediately following thawing (Angel et al. 2016). Therefore, in this study, with a culture period of four hours and a measured viability of T cells after isolation between 70 and 87%, it can be reasonably assumed that a significant decrease in functionality and viability will not occur.

The sample size of the patient with HCC cohorts is relatively small and has an exploratory character. Consequently, small study populations increase the risk of a type II error, whereby an effect is not recognized, and of a type I error, whereby an effect is incorrectly identified as existing when it does not. The overall liver resection cohort included patients with other malignancies, which may affect the generalizability of our findings. Therefore, future studies should aim to validate these observations in larger, more homogenous patient populations. Lastly, animal models could be instrumental in elucidating the functional impact of CD203a expression on Th17 cells and the role of eNAD^+^ in modulating immune responses in the context of HCC. Functional experiments in vivo may provide more definitive evidence of causality and reveal potential therapeutic interventions targeting the CD203a-NAD^+^ axis. The selection of the control group presents a limitation. Patients undergoing hernia surgery were chosen primarily for their availability; however, their liver function and immune profiles may differ significantly from those of patients undergoing liver resection. To better account for the hepatic context, theoretically future studies should consider using patients with benign liver conditions, such as hepatic hemangiomas, as a more appropriate control group.

## Conclusion

The data indicate that eNAD^+^ levels decrease in patients with liver fibrosis/cirrhosis and are correlated with the expression of the ectoenzyme CD203a on Th17 cells. Consequently, patients with high expression of CD203a on Th17 cells had a significantly increased likelihood of recurrence, indicating their potential as valuable prognostic markers and as a target for future therapies.

## Supplementary Information

Below is the link to the electronic supplementary material.

Supplementary file1 (DOCX 1512 KB)

## Data Availability

No datasets were generated or analysed during the current study.
