# Supplementary material for: Extracellular NAD+ levels are associated with CD203a expression on Th17 cells and predict long-term recurrence-free survival in hepatocellular carcinoma
Source: J Cancer Res Clin Oncol. 2025 Mar 19;151(3):115. doi: 10.1007/s00432-025-06155-4 (PMC11923025; doi:10.1007/s00432-025-06155-4)
Supplement: Supplementary file 1 — Supplementary file1 (DOCX 1512 KB) [file 432_2025_6155_MOESM1_ESM.docx]

**Supplements**

**Table I:** patient data and cohorts

| **Characteristics** | **Liver resection group** | | **Control group** |
| --- | --- | --- | --- |
| **Age on the day of surgery (Years)** | M = 62.8 (26-86) | | M = 59 (29-81) |
| **Sex** |  | |  |
| : Female | 36.8% (35) | | 41.7% (10) |
| : Male | 63.2% (60) | | 58.3% (14) |
| **Body Mass Index (kg/m²)** | M = 26.4 (17.3-40.3) | | M = 28.3 (23.1-39.9) |
| : Underweight (BMI = <18.5 kg/m^2^) | 3.2% (3) | | 0% (0) |
| : Normal (BMI = 18.5 - <25 kg/m^2^) | 26.3% (25) | | 16.0% (4) |
| : Overweight (BMI < 25 kg/m^2^) | 56.8% (54) | | 70.8 % (17) |
| : unidentified | 13.7% (13) | | 16.0% (4) |
| **Surgery** |  | |  |
| : abdominal hernia | - | | 45.8 % (11) |
| : other | - | | 54.1 % (13) |
| **Type of resection (Brisbane Criteria)** |  | | - |
| : Exploration, no resection | 15.8% (15) | | - |
| : Minor Resection | 32.6% (31) | | - |
| : Major Resection | 51.6% (49) | | - |
| **Grade of fibrosis (Desmet)** |  | | - |
| : No fibrosis | 10.5% (10) | | - |
| : Grade 1 | 47.3% (45) | | - |
| : Grade 2 | 13.6% (13) | | - |
| : Grade 3 | 11.6% (11) | | - |
| : Grade 4 | 16.8% (16) | | - |
| **Grade of steatosis (%)** | M = 20,8 (0-70) | | - |
| **Entity** |  | | - |
| : Hepatocellular Carcinoma (HCC) | 26.3% (25) | |  |
| : Primary Tumor stage (AJCC-8 ) | T1a | 10% (2) | - |
|  | T1b | 35% (7) | - |
|  | T2 | 20% (4) | - |
|  | T3 | - | - |
|  | T4 | 5% (1) | - |
|  | unknown | 30% (6) | - |
| : Number of Tumors | 1 | 80% (16) | - |
|  | 2 | 15% (3) | - |
|  | 3 | 5% (1) | - |
| : Tumor volume (mm^3^) | M = 437647  (1436 - 1288249) | | - |
| : Living donor transplant | 3.2% (3) | | - |
| : Intrahepatic Cholangiocellular Carcinoma (iCC) | 12.6% (12) | | - |
| : Extrahepatic Cholangiocellular Carcinoma (Klatskin Tumor - eCC) | 11.6% (11) | |  |
| : Metastasis of |  | |  |
| : Colorectal Cancer (CRLM) | 48.4% (46) | | - |
| : Adrenocortical Cancer | 1.1% (1) | Others % (31) | - |
| : Anal Cancer | 1.1% (1) |  | - |
| : Breast Cancer | 1.1% (1) |  | - |
| : Gastrointestinal Stromal Tumor (GIST) | 2.1% (2) |  | - |
| : Granulosa Cell Tumor | 1.1% (1) |  | - |
| : Leiomyosarcoma | 1.1% (1) |  | - |
| : Liposarcoma | 1.1% (1) |  | - |
| : Neuroendocrine Tumor (NET) | 1.1% (1) |  | - |
| : Neuroendocrine Cancer (NEC) | 3.2% (3) |  | - |
| : Non-Small Cell Lung Cancer (NSCLC) | 1.1% (1) |  | - |
| : Stomach Cancer | 1.1% (1) |  | - |
| : Caroli Syndrome | 3.2% (3) |  | - |
| : Cavernous Hemangioma | 2.1% (2) |  | - |
| : Hemangiosarcoma | 1.1% (1) |  | - |
| : Focal Nodular Hyperplasia (FNH) | 1.1% (1) |  | - |
| : Alcohol Related Liver Disease (ArLD) | 1.1% (1) |  | - |
| : Hepatitis C Virus (HCV) | 8.4% (8) |  | - |
| : Primary Sclerosing Cholangitis (PSC) | 1.1% (1) |  | - |
| **Pre-operative LiMAX*** |  | | - |
| : Normal liver function (>315 µg/kg/h) | 55.8% (53) | | - |
| : Impaired liver function (<315 µg/kg/h) | 26.3% (25) | | - |
| : Undefined | 17.9% (17) | | - |
| **Posthepatectomy liver failure (PHLF Classification)** | 10.5% (10) | | - |

(*) LiMax cut-off value defined as >315 µg/kg/h according to Stockmann et al. [(35)](https://paperpile.com/c/ko0cda/WlOj).
